# Infectious Diseases of the Nervous System and Their Impact in Developing Countries

**DOI:** 10.1371/journal.ppat.1000199

**Published:** 2009-02-27

**Authors:** Roberto Bruzzone, Monique Dubois-Dalcq, Georges E. Grau, Diane E. Griffin, Krister Kristensson

**Affiliations:** 1 Hong Kong University-Pasteur Research Centre, Hong Kong, Hong Kong Special Administrative Region, China; 2 National institute of Neurological Disorders and Stroke, National Institutes of Health, Bethesda, United States of America; 3 Department of Pathology, University of Sidney & Bosch Institute, Sydney, Australia; 4 Department of Molecular Microbiology and Immunology, Johns Hopkins Bloomberg School of Public Health, Baltimore, Maryland, United States of America; 5 Department of Neuroscience, Karolinska Institutet, Stockholm, Sweden; The Fox Chase Cancer Center, United States of America

Infectious diseases of the nervous system in the developing world have been relatively neglected. This is paradoxical because neurotropic pathogens are common and contribute significantly to human suffering and disease burden in these regions. Clearly, living with a neurological handicap and/or cognitive dysfunction may have a strong negative impact on socioeconomic development. Here, we briefly describe a few examples of such infections causing severe diseases that result in the loss of motor-sensory function (cerebral malaria, viral encephalitis), alterations of cognition/behavior (AIDS), or sleep perturbations (African trypanosomiasis). Importantly, there is an opportunity to interfere with infection because the central nervous system (CNS) is not usually the primary site for pathogen infection.

## Cerebral Malaria

Cerebral malaria (CM) is one of the most severe complications of *Plasmodium falciparum* malaria. It is most common in young children living in malaria-endemic sub-Saharan Africa where CM incidence is 1–12 cases/1,000 children per year and the mortality rate can be as high as 22%, as described recently in a large cohort of Kenyan children (<14 years old) [1]. Malaria was found to be associated with neurological involvement on admission in nearly half of the patients (with an incidence of 47.6%), and their mortality was increased when compared to malaria patients without neurological signs. The main clinical features consist of seizures often preceding deep coma resulting from cerebral edema, microhemorrhages, and ischemia. Erythrocytes containing malaria parasites accumulate in brain microvessels where leucocytes and platelets are also found.

The multi-factorial complexity of this syndrome has been related to the parasite's release of glycosylphosphatidyl-inositol, which binds to pattern recognition receptors, triggering an inflammatory response and cytokine/chemokine release. TNFα upregulates the endothelial intercellular adhesion molecule ICAM, enhancing binding of parasitized erythrocytes to vascular endothelia with eventual disruption of the blood–brain barrier (BBB) [2]. This may result in activation of microglial cells and astrocytes, demyelination, and/or neuronal injury [3]. Important insights have come from clinical studies, post-mortem analyses of brains from CM victims, in vitro studies of the adhesion of parasitized erythrocytes to brain endothelial cells, and genetic studies of susceptibility and resistance determinants in mice and humans [3]. Balanced views on other aspects of CM pathogenesis and pathophysiology, including metabolic acidosis and capillary dysfunction, have been discussed by Idro et al. [1], who proposed renaming CM as “malaria with neurological involvement”, which leads to long-term neurological sequels and/or behavioral problems in 24% of cases, imposing a major burden on African children.

Although CM is associated with a dramatic activation of brain endothelial cells, with increased expression of ICAM (see [2],[3] for review), remarkably it does not exhibit perivascular infiltrates, and no transendothelial migration of leucocytes occurs. Thus, in CM, inflammation and immune-mediated events remain essentially intravascular, in contrast to other neuroimmunological disorders, such as multiple sclerosis, which is characterized by perivascular infiltrates and no intravascular sequestration of leucocytes. Furthermore, the *Plasmodium*-infected erythrocytes also remain intravascular, in contrast to the direct CNS invasion by other pathogens, such as in toxoplasmosis. Consequently, unless marked hemorrhages occur, there is a limited involvement of parasites or of leucocytes within the CNS parenchyma itself. It is not possible, however, to draw conclusions about a lack of inflammatory pathogenesis, because most, if not all, brain pathology is mediated by intravascular inflammatory events. Concerted interdisciplinary actions are needed to reach a better understanding of CM pathogenesis and the intricate roles of parasite-derived toxins, proinflammatory cytokines, and adhesion molecules. The discovery of increased numbers of endothelial cell membrane–derived microparticles in the circulation during acute CM raises the question of their pathogenic role, as suggested by disease protection in mice lacking one of the genes controlling microparticle formation [4].

Deciphering the precise host immune responses associated with micro-environmental alterations leading to CM is crucial for devising novel therapeutic strategies. Treatment compounds need to be simple, cheap, stable, and easy to handle, such as those recently described in model systems [5],[6]. Current treatment options tackle the parasite but not the host response. Since immunopathology participates in the pathogenesis of CM, new therapeutic approaches, aiming at a down-modulation of excessive host responses, are desirable.

## Human African Trypanosomiasis

Human African trypanosomiasis (HAT), or sleeping sickness, is caused by subspecies of *Trypanosoma brucei* (*Tb*) transmitted by tsetse flies. Although it was nearly eliminated by the 1960s,, HAT has re-emerged as an important threat, with 37,385 reported cases in sub-Saharan Africa in 1998 [7]. However, the estimated number of cases may be ten times as many, although the number of newly detected cases has been declining during the last decade. The West African form is caused by *Tb gambiense*, with humans as the primary host and main reservoir, while the East African form is caused by *Tb rhodesiense*, with wild game or cattle as primary hosts. Both diseases are lethal when untreated, but their temporal profile differs; the West African form lasts several months or even years, whereas the East African form progresses more rapidly [8]. The widely used card agglutination test for screening of trypanosomiasis is only applicable to *Tb gambiense* infections but is not sufficiently sensitive or specific to be diagnostic [7].

How do the parasites cross the BBB and how does this passage relate to efficacy of drug treatment? Can parasites “hide” in the brain parenchyma behind the BBB before relapses occur in unsuccessfully treated patients? How do these extracellular parasites cause the severe neurological symptoms that are manifested most conspicuously in disrupted sleep patterns [9]? Are brain dysfunctions the cause of death in HAT? These questions are important for treatment. Although drugs are relatively effective in curing both forms of HAT early in infection, at later stages, when most patients seek medical help, only drugs that can have severe toxic side effects, such as the widely used arsenic compound melarsoprol, are available. There is, therefore, an urgent need for improving surveillance and diagnostic tools to identify early HAT infection in the field, as well as for new, nontoxic drugs designed to cure brain infections. These issues can be addressed by characterizing the molecules involved in trafficking of trypanosomes across the BBB and the effects on brain functions of molecules released during parasite–immune cell interactions. The strategy of “new use for old drugs”, i.e., prescriptions for neglected diseases of drugs marketed for other illnesses [10], should also be pursued to identify less toxic drugs to cure brain infections.

A new sensitive, specific, and affordable diagnostic test is needed for use in countries where HAT is endemic. Early detection and treatment with better drugs, in combination with improved vector control, will markedly reduce, if not eliminate, the most prevailing West African form of HAT.

## Human American Trypanosomiasis

For *Trypanosoma cruzi*, which causes American trypanosomiasis or Chagas disease, there have been major advances in its control in most Latin American countries where this infection was endemic in the past 20 years. This has been achieved by large-scale programs to halt transmission by eliminating populations of insect vectors (the blood-feeding “assassin bugs”, sub-family *Triatomina*e), screening of blood donors and Chagasic mothers. According to the World Health Report 2004 [11], the disability adjusted life years for Chagas disease were 667,000 and the number of yearly deaths was 14,000 (compared with estimates for HAT of 1,525,000 and 48,000, respectively). Non-treated, the disease evolves in a slow and progressive way into autonomic system neuropathies causing fatal cardiomyopathy and megacolon syndromes in about 30% of the patients. Involvement of the CNS is infrequent, discrete, and non-specific, but in AIDS patients, meningo-encephalitis with parasites in glial cells has been documented [12]. A parasite-derived neurotropic factor that binds to the high-affinity receptor for nerve growth factor TrkA (tropomyosin-related kinase A) may be involved in the pathogenesis of nervous system infection [13]. Reaching an international consensus on diagnostic and treatment procedures is a current priority.

## HIV-Associated Dementia and Neurocognitive Disorders

HIV can spread to the CNS during early and late disease stages, leading to HIV-associated dementia (HAD) or HIV-associated neurocognitive disorders (HAND). In the Western world, where HIV clade B dominates, the advent of highly active anti-retroviral therapy has reduced the incidence of HAD and HAND by approximately half (<15%). The neurobiological basis of these conditions is not due to direct HIV infection of neurons, but to synapto-dendritic alterations called “beading” [14]. Cortical motoneurons and interneurons that show these alterations may eventually die of apoptosis.

In sub-Saharan Africa, where AIDS is prevalent, HAD incidence was studied recently using an accurate, cross-cultural HAD scale [15]. In Uganda, where clades D and A dominate, 31% of AIDS patients develop HAD, which can affect verbal memory, fine and gross motor performances, psychomotor speed, and executive functions. Affected individuals have a higher rate of unemployment than controls and show poor performance in daily family life. In contrast, in Ethiopia where HIV clade C dominates, only minor cognitive alterations were reported in AIDS patients. The lesser impact of HIV on cognitive functions in this case could be explained by the poor ability of HIV clade C isolates to grow in macrophages, a characteristic of neurotropic strains.

Some envelope glycoprotein variants (named N283) are more frequently isolated from infected brains than from other organs [16]. These variants were likely selected by their ability to bind to low levels of the HIV receptor CD4 and co-receptor CCR5 on perivascular macrophages and microglia residing in the CNS. Viral replication in these target cells results in the formation of multinucleated cells. Such giant cells also produce virus that will further spread and persist in the brain where HIV protease inhibitors have limited accessibility. Clearing such a viral reservoir would require specific inhibitors for neurotropic variants that can cross the BBB [17].

More epidemiological studies of HAD and HAND should determine the impact of HIV infection on brain function in different regions of sub-Saharan Africa. To decrease the negative impact of these syndromes on patient cognitive behavior and improve societal acceptance of individuals with HAD or HAND, one needs to isolate and characterize neurotropic viral subtypes from the cerebrospinal fluid early in AIDS. This might allow designing ways to block HIV entry into the CNS.

## Emerging Infectious Diseases of the CNS

Viral encephalitis is emerging or re-emerging as an important cause of human disease due to increased geographic range (e.g., West Nile virus, Japanese encephalitis virus) or new spread of viruses from animal reservoirs into human populations (e.g., Nipah and Hendra viruses) [18],[19]. According to the World Health Organization, Japanese encephalitis is the leading cause of viral encephalitis in Asia, with an annual incidence of 30,000–50,000 clinical cases. Changes in vector populations and in human association with reservoir hosts, and the appearance of new viral variants that are more virulent for humans or more efficiently transmitted, are associated with emerging viruses, half of which cause serious neurological diseases [20].

Arthropod-borne viruses have been restricted in range geographically by the availability of their invertebrate and vertebrate hosts. However, modern transportation has introduced vectors that efficiently transmit arboviruses into new areas (e.g., the Asian tiger mosquito *Aedes albopictus* into North America and Europe). In many areas, pre-existing populations of competent vectors set the stage for successful establishment of viruses in new regions. Recent examples are the introduction of West Nile virus into North America, where susceptible vectors and hosts were abundant and rapid spread across the continent has resulted in more than 11,000 cases of CNS disease, and the arrival of a strain of Chikungunya virus adapted to *Ae. albopictus* in southern Europe. West Nile virus has the widest distribution of all flaviviruses, its range spanning Africa, North, Central, and South America, West Asia, Europe, the Middle East, and Australia [18]. Japanese encephalitis and Rift Valley fever viruses could easily follow the same pattern.

Bats are increasingly recognized as important hosts for a number of zoonoses that cause CNS infection (e.g., lyssaviruses, henipaviruses, coronaviruses, and filoviruses). Disruption of the environment with changing agricultural practices has increased the likelihood that these viruses will be transmitted to humans, as suggested by Nipah and Hendra virus outbreaks. New outbreaks of Nipah encephalitis, nine of which have occurred in Bangladesh since 2001, resulting in the death of 40%–75% of infected people, indicate human-to-human as well as bat-to-human transmission [21].

There are currently no treatments available for these viral CNS diseases. Development of antiviral agents may be useful, but treatment at the time of symptoms may not be effective. Vaccines are likely to be the most effective interventions and are available or in development for many of these viruses (viz., Japanese encephalitis, tick-borne encephalitis, and West Nile); however, to be effectively utilized, spread of the virus must be monitored and disease outbreaks anticipated.

## Perspectives

There is an urgent need for continued surveillance and identification of viruses in vertebrate and invertebrate hosts to anticipate the introduction and spread of new and old agents. A better understanding of the mechanisms of entry into the CNS, of neuronal damage, the immune response to virus infection, and prevention of CNS infection will guide the development of appropriate interventions.

The questions raised here are part of a broader field of investigations on a dozen neurotropic pathogens that were discussed at a September 2008 conference in Paris, “Infections of the Nervous System: Pathogenesis and World Impact”. This conference has addressed the current gaps in knowledge and set the stage to establish an agenda for confronting this group of diseases in the coming years. Abstracts have been published in *BMC Proceedings* ([22]; http://www.biomedcentral.com/1753-6561/2?issue=S1). We need to increase the awareness of the world's leading institutions on the impact and challenges in this field and to foster new research and training programs that will trigger new ideas to study the mechanisms of pathogen spreading and neural cell dysfunction in close contact with clinical research and surveillance, diagnosis, and treatment of infectious neurological diseases. Progress will depend on the development of a systemic approach based on cross-fertilization between clinicians studying disease mechanisms and scientists working on the life cycle and molecular makeup of neurotropic infectious agents and their vectors; between immunologists studying innate and adaptive immune responses to neurotropic pathogens and cell biologists investigating pathogen interactions with the BBB and neural cells; and between leaders in new technologies for diagnosis and therapies and medical anthropologists.
